# A Retrospective Study on the Epidemiology of Anthrax, Foot and Mouth Disease, Haemorrhagic Septicaemia, Peste des Petits Ruminants and Rabies in Bangladesh, 2010-2012

**DOI:** 10.1371/journal.pone.0104435

**Published:** 2014-08-07

**Authors:** Shankar P. Mondal, Mat Yamage

**Affiliations:** Food and Agriculture Organization of the United Nations, Dhaka, Bangladesh; Banaras Hindu University, India

## Abstract

Anthrax, foot and mouth disease (FMD), haemorrhagic septicaemia (HS), peste des petits ruminants (PPR) and rabies are considered to be endemic in Bangladesh. This retrospective study was conducted to understand the geographic and seasonal distribution of these major infectious diseases in livestock based on data collected through passive surveillance from 1 January 2010 to 31 December 2012. Data analysis for this period revealed 5,937 cases of anthrax, 300,333 of FMD, 13,436 of HS, 247,783 of PPR and 14,085 cases of dog bite/rabies. While diseases were reported in almost every district of the country, the highest frequency of occurrence corresponded to the susceptible livestock population in the respective districts. There was no significant difference in the disease occurrences between districts bordering India/Myanmar and non-border districts (p>0.05). Significantly higher (p<0.01) numbers of anthrax (84.5%), FMD (88.3%), HS (84.9%) and dog bite/rabies (64.3%) cases were reported in cattle than any other species. PPR cases were reported mostly (94.8%) in goats with only isolated cases (5.2%) in sheep. The diseases occur throughout the year with peak numbers reported during June through September and lowest during December through April, with significant differences (p<0.01) between the months. The annual usages of vaccines for anthrax, FMD, HS and PPR were only 7.31%, 0.61%, 0.84% and 11.59% of the susceptible livestock population, respectively. Prophylactic vaccination against rabies was 21.16% of cases. There were significant differences (p<0.01) in the administration of anthrax, FMD and HS vaccines between border and non-border districts, but not PPR or rabies vaccines. We recommend that surveillance and reporting of these diseases need to be improved throughout the country. Furthermore, all suspected clinical cases should be confirmed by laboratory examination. The findings of this study can be used in the formulation of more effective disease management and control strategies, including appropriate vaccination policies in Bangladesh.

## Introduction

Bangladesh, located in South Asia, is one of the most densely populated countries in the world with an estimated 1,033 people/km^2^ [1]. It also has the highest density of livestock (cattle, goats, sheep and buffaloes) in the world with an estimated 145 large ruminants/km^2^ compared with 90 for India and 20 for Brazil [2]. Despite declining acreage of pasture land, the livestock population is growing steadily. Livestock are an integral component of agriculture in Bangladesh that includes the provision of draft power and manure. About 20% of the human population is directly and 50% is partly dependent on the livestock sector [3]. Bangladesh has an estimated 52.8 million livestock, mostly food producing cattle and goats [4]. Despite the significant livestock population, the current production of meat and milk is inadequate to meet current requirements of the human population and respective deficits have been estimated at 85.9% and 73.1% [5]. Infectious diseases are of critical importance in livestock production. Economic losses are attributable to decreased animal growth and productivity as well as frequent death of affected animals. Important diseases are foot and mouth disease (FMD), haemorrhagic septicaemia (HS), anthrax in large ruminants (cattle) and peste des petits ruminants (PPR) in small ruminants. Rabies has only begun to be addressed as a major public health threat.

FMD is one of the most important animal diseases currently causing severe economic losses in the South Asian region [6]. FMD, caused by a member of the *Aphthovirus* genus, family *Picornaviridae* [7], is an extremely contagious, acute viral disease primarily of all cloven-hoofed animals [8]. Annual losses due to FMD in Bangladesh are estimated at about US$62 million [9]. Recently, the country has confirmed outbreaks of FMD virus types O and A which are closely related to virus types active in India and Nepal [10], [11]. PPR is usually a disease of small ruminants [12], caused by a member of the *Morbillivirus* genus, family *Paramyxoviridae* [13]. PPR was first isolated in Bangladesh during a major occurrence in 1993 [14]. Since then, the disease has been recognized as endemic in goat. Outbreaks can be associated with up to 75% morbidity and 55% mortality in Black Bengal goats [15]. HS (caused by *Pasteurella multocida*) is another economically important bacterial disease of cattle and buffaloes [16] causing high rates of morbidity and mortality. HS, together with anthrax and black quarter (BQ; not included in this study), are responsible for an estimated economic loss of US$148.6 million each year [17]. Anthrax (caused by *Bacillus anthracis*) is endemic in South Asian countries including Bangladesh [18], [19], [20]. An anthrax outbreak occurred in 2009–2010 resulting in a serious problem in animals and humans; this led to the government declaring a “red alert” in 2010 [21]. Rabies, caused by a member of the *Lyssavirus* genus, family *Rhabdoviridae*, is transmitted mostly (>90%) by the bite of infected dogs to humans [22] and domestic animals [23] in Bangladesh, as in other Asian countries [24]. Bangladesh ranks third in the world after India and China in the number of rabies cases of livestock and/or humans [25]. Bangladesh continues to experience outbreaks of these diseases in livestock despite vaccination (except rabies) as part of a government effort to control them. Major factors to account for the continuing problem include inadequate monitoring, surveillance and disease reporting, lack of public awareness, lack of any controls over animal movements, inadequate management and vaccination strategies.

To effectively combat the threats posed by the selected and other diseases, there is a need for clear understanding of the epidemiology of the respective diseases [26]. The goal of an epidemiological study is to identify risk factors that need to be taken into consideration in the development of effective control measures. Information on the incidence of major infectious diseases (e.g., FMD) in livestock is available from a number of countries in which the diseases have been reported [27], [28]. While there have been some studies of the incidence and distribution of FMD [29], [30], PPR [31], HS [32], [33], anthrax [19], [20] and dog bite/rabies cases [23], [33] in livestock in Bangladesh, the geographic areas and duration involved in such studies are too limited to reflect more accurately the national status. The Food and Agriculture Organization of the United Nations (FAO) provided technical assistance to the Government of Bangladesh (GoB) to undertake an analysis using passive surveillance data of the overall disease situation in livestock in the country. Accordingly, this study was conducted to investigate the geographic and seasonal distributions of anthrax, FMD, HS, PPR and dog bite/rabies in livestock throughout the country, based on available passive surveillance data for the period January 2010 to December 2012.

## Methods

### Geography of the study area

**Figure 1:**
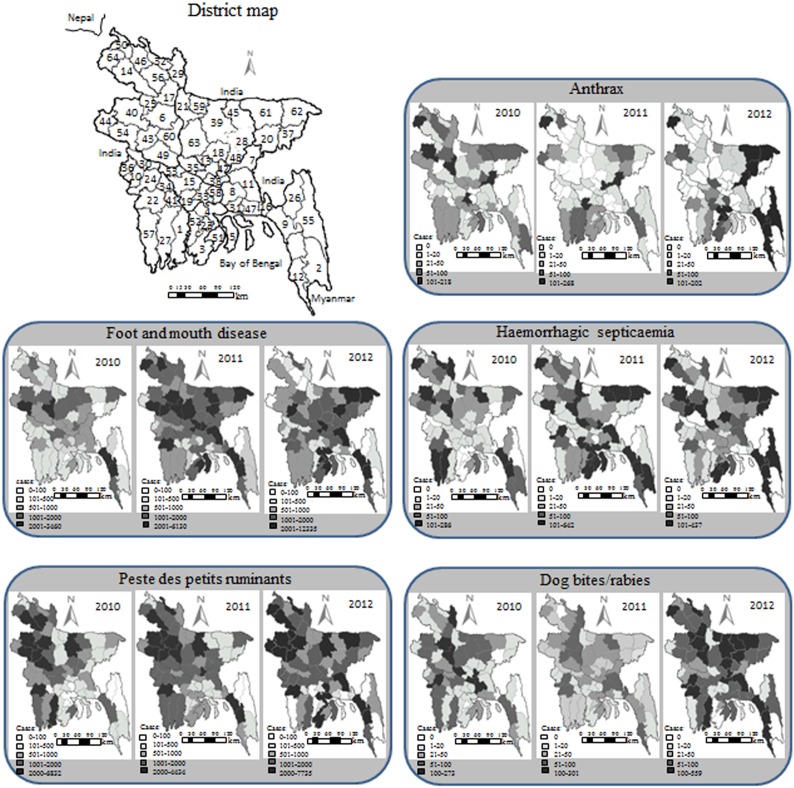
Map of Bangladesh showing 64 districts (top left): 1) Bagerhat, 2) Bandarban, 3) Barguna, 4) Barisal, 5) Bhola, 6) Bogra, 7) Brahmanbaria, 8) Chandpur, 9) Chittagong, 10) Chuadanga, 11) Comilla, 12) Cox’s Bazar, 13) Dhaka (the capital), 14) Dinajpur, 15) Faridpur, 16) Feni, 17) Gaibandha, 18) Gazipur, 19) Gopalganj, 20) Habiganj, 21) Jamalpur, 22) Jessore, 23) Jhalokathi, 24) Jhenaidaha, 25) Joypurhat, 26) Khagrachhari, 27) Khulna, 28) Kishoreganj, 29) Kurigram, 30) Kushtia, 31) Laksmipur, 32) Lalmonirhat, 33) Madaripur, 34) Magura, 35) Manikganj, 36) Meherpur, 37) Moulvibazar, 38) Munshiganj, 39) Mymensingh, 40) Naogaon, 41) Narail, 42) Narayanganj, 43) Natore, 44) Nawabganj, 45) Netrokona, 46) Nilphamari, 47) Noakhali, 48) Norsingdi, 49) Pabna, 50) Panchgarh, 51) Patuakhali, 52) Pirojpur, 53) Rajbari, 54) Rajshahi, 55) Rangamati, 56) Rangour, 57) Satkhira, 58) Shariatpur, 59) Sherpur, 60) Sirajganj, 61) Sunamganj, 62) Sylhet, 63) Tangail, 64) Thakurgaon. The maps illustrate district distribution of the estimated number of diagnosed cases of anthrax, foot and mouth disease, haemorrhagic septicaemia, peste des petits ruminants and dog bite/rabies cases reported in livestock (cattle, goats, sheep and buffaloes) in the country, 2010–12.

Bangladesh has a unique geographical location with an area of 147,570 km^2^. The country is exceedingly flat, low-lying and subject to annual flooding, except for the narrow hilly regions in the northeast and southeast. It has one of the largest deltas of the world formed by two main rivers, Padma (Ganges in India) and Jamuna (Brahmaputra in India), which become confluent as a single river (Padma). The country has land boundaries totaling 4,246 km, of which the vast majority are with India and only 193 km at the southeastern border are with Myanmar (Figure 1). The existence of formal and informal cross-border movement of animals, animal products and feed is a traditional practice among the countries in the region. Bangladesh is divided into 7 divisions, 64 districts and 487 sub-districts or upazilas (an upazila is a lower administrative unit in the country). The livestock population is approximately 52.8 million, consisting of 23.2 million cattle, 1.4 million buffaloes, 25.1 million goats and 3.1 million sheep [4]. The majority of livestock are reared by smallholders in integrated agricultural farming systems.

### Data source

For this study, passive surveillance animal disease data at the upazila level were determined from January 2010 to December 2012 (both months inclusive). Data were obtained from the Epidemiology Unit of the Department of Livestock Services (DLS), Dhaka, Bangladesh. The latter unit was established with technical support from the FAO after highly pathogenic avian influenza (HPAI) H5N1 virus was detected in the country in 2007. Bangladesh has a government-financed veterinary network that extends to the upazila level where it is headed by the Upazila Livestock Officer (ULO). Clinical records are kept at the upazila veterinary hospitals for all cases treated by staff veterinarians. Usually, animals seen by clinicians are sick animals presented by farmers. ULOs send monthly passive surveillance reports to the district level office (DLO), which in turn are sent to the Epidemiology Unit of DLS. This unit is responsible for carrying out outbreak investigations and specimen collection, compilation and analysis of disease reports. The current study was based on analysis of monthly reports for 2010 from 418 upazilas of 62 of the 64 districts (74% upazila coverage), for 2011 from 478 upazilas of all 64 districts (87% upazila coverage) and for 2012 from 471 upazilas of 63 districts (91% upazila coverage). No disease reports were available from Rangamati and Feni districts in 2010 and from Bandarban district in 2012.

For the purpose of this study, a case is defined as an animal with a history, clinical signs or lesions characteristic of anthrax, FMD, HS, PPR or dog bite/rabies; in some instances, there was supportive laboratory data from examination of pathological samples [34], [35]. The term “dog bite” is used for a suspected rabies case as every animal bite (mostly dog bite) is potentially suspected as coming from a rabid animal. The signs/lesions are sufficient for registered veterinarians to make a provisional diagnosis of the endemic diseases in Bangladesh. Demographic data, such as date (month) animal was seen by clinicians, owner’s address (upazila, district and division), animal species involved, disease diagnosed and utilization of vaccine were included in the reports. To avoid duplication with number of animals treated (especially in case of follow up visits), the status of the diseases is described using only newly reported cases.

### Data analysis

#### Descriptive and temporal analysis

The data, obtained from DLS in Microsoft Access database, were transferred to a spreadsheet program (Excel 2007, Microsoft) (spreadsheet is available on request). Descriptive statistical analysis was conducted to examine the frequencies and annual patterns of anthrax, FMD, HS, PPR and dog bite/rabies occurrences. Three years’ (2010 to 2012) data were aggregated into monthly estimated number of diagnosed cases, death cases and vaccination coverage against each disease; time series plots were created for each disease to visualize possible trends and seasonality. Each year was divided into the four main weather seasons in Bangladesh [36]: (1) pre-monsoon (March to May), (2) monsoon (June to August), (3) post-monsoon (September to November) and (4) winter (December to February). The seasonal distribution was assessed by summing the frequency of cases into these four seasons. The estimated number of diagnosed cases was calculated under a null hypothesis that each disease report was independent of particular time of the year. Observed and expected numbers were computed by Chi-square test using GraphPad Software (www.graphpad.com/quickcalcs/index.cfm) to evaluate the association between the disease report and time of the year. Analysis of level of significance was conducted for diagnosed/death cases per 1,000 animals examined and for vaccination rates per 10,000 susceptible animals.

#### Spatial analysis

Adobe Fireworks CS5.1 software program (Adobe Systems Inc., 345 Park Avenue San Jose, CA) was used to create maps for district distribution of the diseases in livestock in Bangladesh. Available case reports in any species of livestock (all ruminants for anthrax, FMD, HS and dog bite/rabies, and small ruminants for PPR) were aggregated at the district level and demonstrated in the map for yearly distribution of each disease (Figure 1). Since these diseases were reported from most of the districts of Bangladesh, the frequencies of cases in each district were presented separately for three years (2010 to 2012) to describe the annual distribution of reported cases in these districts.

## Results

### Descriptive analysis

From January 2010 through December 2012, records of 4,728,522 clinical cases in livestock (including 2,900,621 cattle, 1,545,831 goats, 141,707 sheep and 139,737 buffaloes) were collected from DLS veterinary clinics through a nation-wide passive surveillance program. Of the clinical diseases under study, the breakdown of numbers of reported cases are anthrax (5,937), FMD (300,333), HS (13,436), PPR (247,783) and dog bite/rabies (14,085) (Table 1). Except for anthrax, the number of reports of other diseases was higher in 2011 or 2012 than 2010. Anthrax cases were reported more in 2010 (n = 2,174) than 2011 (n = 1,668) or 2012 (n = 2,095). The mean reported prevalence rate (based on number of livestock examined) was highest for PPR (14.68%: 95% CI 14.63–14.74%), which was calculated based on the number of goats and sheep examined only, followed by FMD (6.35%: 6.33–6.37%), dog bite/rabies (0.30%: 0.29–0.30%), HS (0.28%: 0.28–0.29%) and anthrax (0.13%: 0.12–0.13%). The case fatality rate (CFR) was highest for cases of dog bite/rabies (24.32%: 95% CI 23.61–25.03%), followed by anthrax (13.49%: 12.62–14.37%), PPR (4.86%: 4.77–4.94%), HS (2.87%: 2.59–3.16%) and FMD (1.31%: 1.27–1.35%). The CFR of anthrax was reported higher in 2010 (19.92%) than in 2011 (10.37%) or 2012 (9.31%). Based on a livestock census conducted in 2005 in Bangladesh [5], reports of vaccination coverage against any of the five diseases were very low. The maximum coverage was against PPR (11.59%: 95% CI 11.58–11.60%) in susceptible small ruminants, followed by anthrax (7.31%: 7.31–7.32%), HS (0.84%: 0.83–0.84%) and FMD (0.61%: 0.60–0.61%) in all susceptible ruminants. Rabies vaccine used as post-exposure prophylaxis and calculated based on the number of total reported dog bite cases was 21.16% (95% CI 20.48–21.84%).

**Table 1:** Estimated number of diagnosed cases, death cases and vaccination coverage of anthrax, foot and mouth disease, haemorrhagic septicaemia, peste des petits ruminants and dog bite/rabies in livestock (cattle, goats, sheep and buffaloes) in Bangladesh, 2010–2012.

Disease	2010	2011	2012	Total
**Anthrax**				
** **Diagnosed cases	2,174	1,668	2,095	5,937
** **Prevalence rate, % (95% CI*)	0.14 (0.13–0.15)δ	0.09 (0.08–0.09)	0.17 (0.16–0.18)	0.13 (0.12–0.13)
Death cases	433	173	195	801
Case fatality rate, % (95% CI)	19.92 (18.23–21.61)	10.37 (8.90–11.84)	9.31 (8.06–10.56)	13.49 (12.62–14.37)
Vaccination	2,602,967	3,417,136	3,325,525	9,345,628
Vaccination rate, % (95% CI)	6.11 (6.10–6.12)†	8.02 (8.01–8.03)	7.81 (7.80–7.82)	7.31 (7.31–7.32)
**Foot and mouth disease**				
Diagnosed cases	44,314	93,968	162,051	300,333
Prevalence rate, % (95% CI*)	2.81 (2.79–2.84)	4.95 (4.92–4.99)	12.91 (12.85–12.97)	6.35 (6.33–6.37)
Death cases	651	1,566	1,721	3,938
Case fatality rate, % (95% CI)	1.47 (1.36–1.58)	1.67 (1.58–1.75)	1.06 (1.01–1.11)	1.31 (1.27–1.35)
Vaccination	253,084	213,654	311,718	778,456
Vaccination rate, % (95% CI)	0.59 (0.59–0.60)	0.50 (0.49–0.50)	0.73 (0.73–0.74)	0.61 (0.60–0.61)
**Haemorrhagic septicaemia**				
Diagnosed cases	2,782	5,885	4,769	13,436
Prevalence rate, % (95% CI*)	0.18 (0.17–0.18)	0.31 (0.30–0.32)	0.38 (0.37–0.39)	0.28 (0.28–0.29)
Death cases	64	221	101	386
Case fatality rate, % (95% CI)	2.30 (1.74–2.86)	3.76 (3.27–4.24)	2.12 (1.71–2.53)	2.87 (2.59–3.16)
Vaccination	272,395	367,862	433,101	1,073,358
Vaccination rate, % (95% CI)	0.64 (0.63–0.64)	0.86 (0.86–0.87)	1.02 (1.01–1.02)	0.84 (0.83–0.84)
**Peste des petits ruminants**				
Diagnosed cases	69,684	78,441	99,658	247,783
Prevalence rate, % (95% CI*)	12.08 (11.99–12.16)	11.27 (11.20–11.35)	24.01 (23.88–24.14)	14.68 (14.63–14.74)
Death cases	4,417	4,094	3,523	12,034
Case fatality rate, % (95% CI)	6.34 (6.16–6.52)	5.22 (5.06–5.38)	3.54 (3.42–3.65)	4.86 (4.77–4.94)
Vaccination	1,514,476	2,127,698	2,427,648	6,069,822
Vaccination rate, % (95% CI)	8.67 (8.66–8.69)‡	12.19 (12.17–12.20)	13.91 (13.89–13.92)	11.59 (11.58–11.60)
**Dog bite/rabies** θ				
Diagnosed cases	2,930	3,904	7,251	14,085
Prevalence rate, % (95% CI*)	0.19 (0.17–0.19)	0.21 (0.20–0.21)	0.58 (0.56–0.59)	0.30 (0.29–0.30)
Death cases	781	1,159	1,485	3,425
Case fatality rate, % (95% CI)	26.66 (25.05–28.27)	29.69 (28.25–31.13)	20.48 (19.55–21.41)	24.32 (23.61–25.03)
Vaccination	579	767	1,634	2,980
Vaccination rate, % (95% CI)	19.76 (18.31–21.21)‡	19.64 (18.39–20.90)	23.54 (21.57–23.50)	21.16 (20.48–21.84)

*CI = Confidence intervals.

δ Calculated based on the number of livestock (cattle, buffaloes, sheep and goats) examined, which is 1,576,562 in 2010; 1,896,815 in 2011; and 1,255,145 in 2012 (only sheep and goats 576,936 in 2010; 695,845 in 2011; and 415,007 in 2012).

† Calculated based on susceptible livestock population (buffaloes, cattle, goats and sheep) in Bangladesh, which is 42,594,399 [5].

‡ Calculated based on goat and sheep population only, which is 17,459,061 [5].

θ The term “dog bite” is used for a suspected rabies case as every animal bite (mostly dog bite) is potentially suspected as rabid animal bite, as the rabies control program in animals is nonexistent in Bangladesh [22].

Calculated based on reported dog bite cases assuming that all death cases are from rabies.

### Geographic distribution

The spatial distribution of reported anthrax, FMD, HS, PPR and dog bite/rabies cases from January 2010 to December 2012 in livestock from different districts of Bangladesh is presented in Figure 1 and Tables S1–S3. Based on the overall data from 2010–2012, of the 487 upazilas in the country, anthrax and HS were reported in 169 (34%) and 186 (38%) upazilas from 61 of the 64 districts, respectively, and FMD, PPR and dog bite/rabies cases were reported in 445 (91%), 401 (82%) and 253 (52%) upazilas from all 64 districts of the country, respectively. The number of anthrax reports was highest in Thakurgaon (10%), Naogaon (8.4%), Bagerhat (7%), Sirajganj (5.1%) and Brahmanbaria (4.9%) districts in 2010; in Thakurgaon (16.1%), Narayanganj (12.3%), Brahmanbaria (8.5%) and Gopalganj (7.5%) districts in 2011; and in Rangamati (9.6%), Sunamganj (8.6%), Sylhet (8.5%), Brahmanbaria (7.4%) and Thakurgaon (6.9%) in 2012 (Figure 1 and Table S1); this accounted for 35.5%, 44.3% and 41.0% of all anthrax cases reported in the respective year. Sirajganj (a milk shed area) reported one of the highest number of anthrax cases in 2010, but this district did not have any reported cases of anthrax in 2011 or 2012. In all three years (2010 to 2012), stable and the highest number of FMD cases were reported in Chittagong, Sylhet, Rajshahi, Tangail, Bogra and Sirajganj districts; this accounted for 29.1%, 17.8% and 13.8% of all FMD cases reported, respectively. The Comilla district reported only a few FMD cases (1.8%) in 2010, but this district reported the highest number of FMD cases in 2011 (5.7%) and 2012 (7.6%). Thakurgaon, Chittagong and Sirajganj districts consistently reported the highest HS cases in all three years; this accounted for 18.8%, 16.7% and 13.9% of all HS cases reported in the respective year. The Barguna district reported none and Patuakhali reported only a few HS cases in 2010, but these two districts reported higher numbers of HS cases in 2011 (16.6%) and 2012 (17.2%). Consistently high PPR cases were reported in Jessore, Rajshahi, Bogra, Mymensingh, Naogaon and Chittagong districts during this three-year period; this accounted for 32.0%, 25.6% and 26.4% of all PPR cases reported, respectively. The number of dog bite/rabies reports was consistently high in Sirajganj, Munshiganj, Jhenaidaha, Bogra, Gaibandha, Narayanganj, Jessore and Chandpur districts in three years; this accounted for 42.5%, 32.9% and 34.9% of all dog bite/rabies cases reported, respectively. The two hilly districts Rangamati and Khagrachhari had the lowest number of disease cases. The annual patterns of the diseases in six upazilas with maximum reported cases are shown in Table S2. In 2010, Ranishankail upazila (Thakurgaon district) reported the highest number of both anthrax (n = 105, 4.83%) and HS (n = 232, 8.34%) cases, and Charghat (Rajshahi), Badarganj (Rangpur) and Lahajang (Munshiganj) reported the highest number of FMD (n = 1,664, 3.76%), PPR (n = 1,391, 2.0%) and dog bite/rabies (n = 240, 8.19%) cases, respectively. In 2011, Sonargaon upazila (Narayanganj district) reported the highest number of both anthrax (n = 183, 10.97%) and PPR (n = 1,459, 1.86%) cases, and Kaliganj (Lalmonirhat) and Patuakhali Sadar (Patuakhali) reported the highest number of FMD (n = 1,493, 1.59%) and HS (n = 373, 6.34%) cases, respectively. In 2012, Belaichhari (Rangamati district), Nalchhiti (Jhalokati), Baraigram (Natore) and Lalmonirhat Sadar (Lalmonirhat) reported the highest number of anthrax (n = 165, 7.88%), FMD (n = 4,830, 2.98%), HS (n = 434, 9.10%) and PPR (n = 2,790, 2.80%) cases, respectively. Shahjadpur upazila (Sirajganj district) reported the highest number of dog bites/rabies cases in both 2011 (n = 213, 5.46%) and 2012 (n = 329, 4.54%). Occurrence of the diseases corresponded to the number of susceptible livestock in the respective districts (Table S1). Further analysis showed that 50% of the districts (32/64) bordering India (31) and Myanmar (1) reported nearly equal numbers of cases compared to reports in non-border districts; there was no significant difference (p>0.05) between them (Table S3).

### Species susceptibility

Table 2 shows the distribution of reported anthrax, FMD, HS, PPR and dog bite/rabies cases in different livestock species (cattle, goats, sheep and buffaloes) in Bangladesh. Data analysis showed that significantly higher (p<0.01) numbers of anthrax (84.5%), FMD (88.3%), HS (84.9%) and dog bite/rabies (64.3%) cases were reported in cattle than any other species. PPR cases were mostly reported in goats (94.8%) with the remainder in sheep (5.2%). The CFR of anthrax and dog bite/rabies were significantly higher (p<0.01) in cattle (15.5% and 31.4%, respectively) than other species (≤3.2% and ≤15.5%, respectively), but the CFR of HS was significantly higher (p<0.01) in buffaloes (7.9%) than cattle (2.0%) or goats/sheep (0%). The difference in the CFR of FMD or PPR between the species was not statistically significant (p>0.05).

**Table 2:** Species distribution of estimated number of diagnosed cases and death cases of anthrax, foot and mouth disease, haemorrhagic septicaemia, peste des petits ruminants and dog bite/rabies in livestock in Bangladesh, 2010–2012.

		2010		2011		2012		Total	
Diseaseδ	Species	Diagnosedcases (%)	Death cases(CFRθ, %)	Diagnosedcases (%)	Death cases(CFR, %)	Diagnosedcases (%)	Death cases(CFR, %)	Diagnosedcases (%)	Death cases(CFR, %)
Anthrax	Buffaloes	67 (3.1)	0	83 (5.0)	3 (3.6)	125 (5.97)	3 (2.4)	275 (4.63)	6 (2.18)
	Cattle	1879 (86.4)*	423 (22.5)*	1278 (76.6)*	165 (12.9)*	1858 (88.69)*	188 (10.12)*	5015 (84.47)*	776 (15.47)*
	Goats	220 (10.1)	10 (4.6)	270 (16.2)	5 (1.9)	112 (5.35)	4 (3.57)	602 (10.14)	19 (3.16)
	Sheep	8 (0.4)	0	37 (2.2)	0	0	0	45 (0.76)	0
FMD	Buffaloes	1015 (2.3)	2 (0.2)	3187 (3.4)	62 (2.0)	4596 (2.84)	4 (0.09)	8798 (2.93)	68 (0.77)
	Cattle	39694 (89.6)*	593 (1.5)	81515 (86.8)*	1277 (1.6)	143953 (88.83)*	1579 (1.10)	265162 (88.29)*	3449 (1.30)
	Goats	3444 (7.8)	56 (1.6)	8662 (9.2)	209 (2.4)	12287 (7.58)	134 (1.09)	24393 (8.12)	399 (1.64)
	Sheep	161 (0.3)	0	604 (0.6)	18 (3.0)	1215 (0.75)	4 (0.33)	1980 (0.66)	22 (1.11)
HS	Buffaloes	215 (7.7)	4 (1.9)	834 (14.2)	110 (13.2)*	907 (19.02)	41 (4.52)	1956 (14.56)	155 (7.92)*
	Cattle	2496 (89.7)*	60 (2.4)	5051 (85.8)*	111 (2.2)	3859 (80.92)*	60 (1.56)	11406 (84.89)*	231 (2.03)
	Goats	66 (2.4)	0	0	0	3 (0.06)	0	69 (0.51)	0
	Sheep	5 (0.2)	0	0	0	0	0	5 (0.04)	0
PPR	Goats	66717 (95.7)*	4229 (6.3)	73066 (93.1)*	3862 (5.3)	95096 (95.42)*	3414 (3.59)	234879 (94.79)*	11505 (4.90)
	Sheep	2967 (4.3)	188 (6.3)	5375 (6.9)	232 (4.3)	4562 (4.58)	109 (2.39)	12904 (5.21)	529 (4.10)
Dog bite	Buffaloes	33 (1.1)	0	32 (0.8)	9 (28.1)	64 (0.88)	11 (17.19)	129 (0.92)	20 (15.50)
/rabies	Cattle	2235 (76.3)*	705 (31.5)	2604 (66.7)*	939 (36.0)	4217 (58.16)*	1201 (28.48)*	9056 (64.30)*	2845 (31.42)*
	Goats	606 (20.7)	69 (11.4)	1133 (29.0)	209 (18.5)	2763 (38.11)	269 (9.74)	4502 (31.96)	547 (12.15)
	Sheep	56 (1.9)	7 (12.5)	135 (3.5)	2 (1.5)	207 (2.86)	4 (1.93)	398 (2.83)	13 (3.27)

δ Abbreviated: FMD = Foot and mouth disease, HS = Haemorrhagic septicaemia, PPR = Peste des petits ruminants.

θ CFR = Case fatality rate.

*Statistically significant at the p<0.01 level.

### Temporal distribution

**Figure 2:**
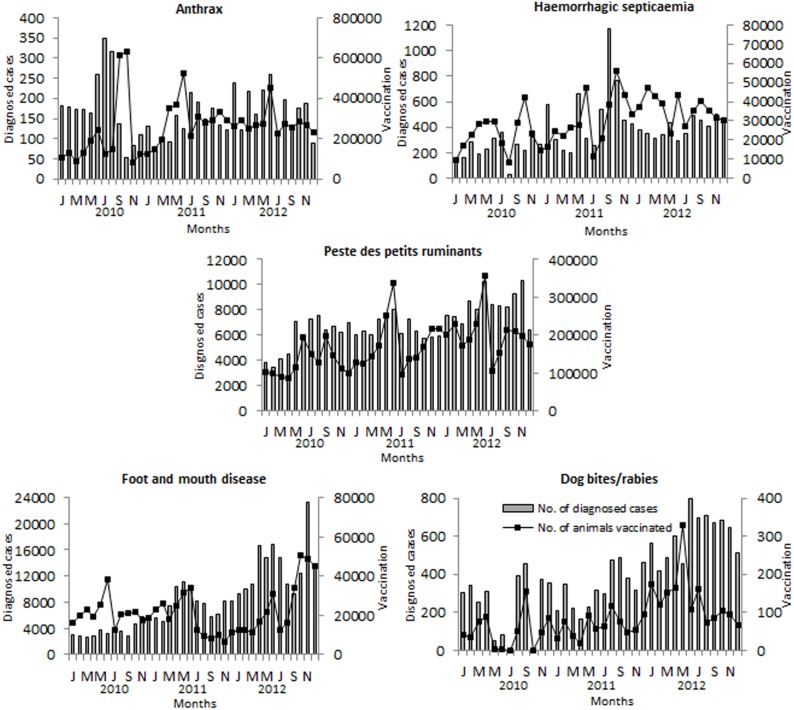
Monthly distribution of animal diseases and vaccination status in Bangladesh, 2010–2012. It represents the estimated number of diagnosed cases and vaccination of anthrax, foot and mouth disease, haemorrhagic septicaemia, peste des petits ruminants and dog bite/rabies reported in livestock (cattle, goats, sheep and buffaloes) in the country.

Monthly and seasonal distribution of anthrax, FMD, HS, PPR and dog bite/rabies reports from January 2010 through December 2012 are presented in Figure 2 and Table S4. All five diseases were reported throughout the year in livestock (Figure 2). The highest number of anthrax cases was reported in the month of August (n = 704, 11.9%) followed by July (11.3%); FMD in November (n = 37,204, 12.4%) followed by June (10.2%); HS in September (n = 1,893, 14.1%); PPR in June (n = 23,962, 9.7%) followed by August (9.3%); and dog bite/rabies in September (n = 1,612, 11.5%) followed by August (11.2%). The lowest number of anthrax, FMD, HS, PPR and dog bite/rabies reports was in December (5.4%), February (5.9%), April (5.4%), March (6.9%) and May (5.2%), respectively. There were significant differences between the months in the reported cases of each disease (p<0.01). When the overall data were grouped into the four main weather seasons in Bangladesh [36] (Table S4), the highest number of anthrax reports was observed in monsoon (34%), FMD in pre-monsoon (26.7%) followed by monsoon (26.3%), HS in post-monsoon (34%), PPR in monsoon (27.8%) and dog bite/rabies in post-monsoon (28.4%) followed by monsoon (26.7%). The lowest number of anthrax, FMD, HS, PPR and dog bite/rabies cases was reported in post-monsoon (20.5%), winter (20.9%), pre-monsoon (21.4%), winter (21.8%) and pre-monsoon (19.6%) season, respectively. There were significant differences (p<0.05) between the seasons on the occurrences of anthrax, HS, PPR and dog bite/rabies, but not FMD (p = 0.13). The highest number of death cases for each disease corresponded to the highest number of cases reported in the respective season. There was no significant differences (p>0.05) in the CFR of anthrax, FMD, PPR or dog bite/rabies between the seasons, but the CFR for HS was significantly different between the seasons (p<0.01).

### Vaccination

Geographic (border *vs.* non-border districts) and monthly distribution of vaccination coverage against anthrax, FMD, HS, PPR and rabies from January 2010 through December 2012 are presented in Figure 2 and Table S3. Based on the 2005 livestock census in Bangladesh [5], there were significant differences (p<0.01) between border and non-border districts on the coverage of anthrax (6.14% and 8.91%, respectively), FMD (0.46% and 0.81%, respectively) and HS (0.69% 1.04%, respectively) vaccines, but not (p>0.05) PPR (12.24% and 10.60%, respectively) or rabies (19.68% and 23.51%, respectively) vaccines (Table S3). Sirajganj district reported the highest (>30%) coverage of anthrax vaccine in livestock in 2010. Significantly higher (p<0.0001) coverage of PPR vaccine was reported in southern Khulna and northern Rajshahi divisions (where the goat population is very high [5]) compared to the national average, which accounted for 16.8% of all vaccinations reported during this three-year period. The highest coverage of anthrax, FMD, HS, PPR and rabies vaccines was in June (13%), June (13.2%), October (12.4%) followed by June (11.2%), June (14.6%) and May (14.3%), respectively, and lowest in January (5.3%), July (4.8%), July (5.3%), July (5.8%) and October (5.1%), respectively (Figure 2).

## Discussion

This is the first retrospective analysis of the geographic and seasonal distribution of anthrax, FMD, HS, PPR and dog bite/rabies cases in livestock in Bangladesh. The data show that i) the diseases occur across the country irrespective of border and non-border districts, but the highest frequency of occurrence corresponded to the number of susceptible livestock in respective districts; ii) most of the reported anthrax, FMD, HS and dog bite/rabies cases are in cattle and PPR in goats, the two predominant livestock species in the country [4]; iii) the diseases occur throughout the year but the highest frequency of occurrence are in the months of June through September (mostly during the monsoon season) and lowest in December through April (mostly during the winter season); and iv) the vaccination coverage against each of the diseases studied is inadequate, especially for anthrax, FMD and HS in the border districts. Therefore, it would be appropriate to target geographic areas and time of the year for future disease surveillance and control programs in the country.

When the reported data were analyzed based on geographic locations, consistently high numbers of cases for all five diseases were reported in Sirajganj, Narayanganj, Bogra, Chittagong, Thakurgaon and Naogaon districts (Figure 1 and Table S1), the latter three all border with India. The noted differences in disease occurrence could be due to high density of animal populations and widespread movement of animals between countries and within the country with mixing where animals are pastured or in animal markets. Sirajganj (a milk shed area) has a history of reporting high clinical cases of FMD (n = 3,708) in 1999 [37]; this district confirmed anthrax almost exclusively during 2009–2010 [19], [20]. Whereas, Narayanganj is a small, densely human populated industrial area close to Dhaka (the capital); this has seen high numbers of cases of rabies during 2004–2008 [22] and in 2010 [38]. Data showed that the ratio of border/non-border cases with anthrax, FMD, HS, PPR or rabies infection was nearly equal (Table S3), with no significant difference between them (p>0.05). However, there is molecular evidence that the FMD virus (types O and A) recently isolated in Bangladesh were closely related to FMD viruses in circulation in India and Nepal [10], [11]. Also, the recent PPR virus strains detected in Bangladesh and the neighboring countries including India, Nepal and Pakistan belong to the same lineage IV [39]. India and Nepal consume less meat especially beef, due to religious reasons. Therefore, the price differences encourage constant informal cross-border animal movement from India and Nepal to Bangladesh, with most animals being destined for the large cities. There are no border controls or animal quarantine [40] between the three countries. Moreover, cross-border movement of free-roaming dogs from India and Myanmar, two highly rabies-endemic countries in the world [41], could explain the persistent, high incidence of rabies in Bangladesh [22]. A considerable number of cattle come from Myanmar also. Several studies have confirmed cross-border animal movements as one of the main reasons for the spread and persistence of FMD [6], [42], HS [16] and PPR [39], [43]. During movement, these animals frequently infect local susceptible animal populations along the transportation routes [44]. The infected animals help to maintain virus circulation throughout the year. Live animal markets are held once or twice a week in most upazilas of Bangladesh. All disease-susceptible farm animals are sold at these markets. Previous studies have demonstrated a correlation between the market chain of livestock and the geographical spread of FMD [26] and PPR [45]. Animals receive minimum preventive veterinary care in Bangladesh, which further increases the risk of acquiring infections.

Cattle were the predominant species affected with anthrax (Table 2), which is consistent with anthrax reports from northwestern Bangladesh during 2009–2010 [19]. Cattle are also known to be highly susceptible to inhalation of aerosolized FMD virus [46] and can become persistently infected [47]. This species was found to account for the highest number of cases of FMD in this study. This is consistent with the FMD epidemiology in Bhutan [28], India [48] and Nepal. Factors responsible may include the fact that cattle are the most populous species of livestock found in Bangladesh; cattle graze in plains (usually in large groups) whereas goats graze in the high lands (in small groups or individually); and, due to the higher economic value of cattle over goats and sheep, sick cattle are more often referred for veterinary attention. Buffaloes are more susceptible to HS than cattle [49], which explains the high CFR of HS in this species (Table 2). The overall low CFR of anthrax in all livestock species could be due to production of immunity by massive vaccination, early diagnosis and treatment of sick animals presented by farmers or the likely biases in the reporting system (see below). Rabies is not a notifiable disease in Bangladesh, so the surveillance data in animals and humans are scarce. A comprehensive study by Hossain et al. [22] determined 732 human rabies cases (0.5%) out of 150,068 animal bite cases treated from 2005 through 2008, most of which were the result of dog bites (90.7%). In our study, 3,425 animal fatalities (24.32%) were reported out of 14,085 dog bites in livestock during 2010–2012. More dog bite/rabies cases were reported in goats than anthrax, FMD and HS cases probably due to more availability and easy access of this species by street dogs in village areas, or possibly more goat owners brought sick animals to the clinics. In Bangladesh, the proportion of goats to sheep is about 8∶1 [4]. In this study, a higher number of PPR cases was observed in goats than sheep (16∶1); this is consistent with the PPR epidemiology in India [44], [45] suggesting that differences in the virulence between species could be due to greater innate resistance in sheep.

In this study, reports of infectious disease occurrences throughout the year (mostly during the monsoon season) are consistent with the previous reports for anthrax [19], FMD [10], [36], HS [33] and animal bite/rabies [22] cases in Bangladesh. Sudden anthrax outbreaks occurred in animals during July–August (monsoon) in 2010 (Figure 2). Environmental factors, including high ambient temperature and relative humidity that provide a milieu for germination of anthrax spores from infected carcasses thrown into flood waters or in open fields [20], may favor the presence of anthrax in Bangladesh [50]. During the rainy season, grass harvested along with roots might also harbor anthrax spores and when fed, livestock can become infected [19]. Moist conditions also prolong the survival of the HS organism (*P. multocida*), and thus the disease tends to spread more rapidly during the monsoon season when rice cultivation also brings about movements of animals [16]. Increased numbers of dog bite/rabies reports during post-monsoon and monsoon may be associated with the breeding season of dogs [51]. A significantly higher number of FMD cases during April–June (pre-monsoon) and November in 2012 (Figure 2) could be due to the increased movement of animals from outside and within the country for trade purposes during this particular year. The movement of livestock for management and husbandry purposes is important for local spread of disease [28]. Stress can also suppress the development of immunity and hence predispose an animal to infection [52]. In this study, more PPR cases in goats and sheep were also reported during the monsoon season (Table S4). This could be due to limited availability of feed during the summer and wet seasons and close confinement of goats in the households during this period of the year [44]. Introduction of new animals into the flocks could be another possibility. The feeding habits of goats are quite different from cattle (see above). Climate factors favorable for the survival and spread of the virus may also contribute to the seasonal distribution of PPR [53]. As opposed to our finding, a local study in Bangladesh reported highest PPR cases in the months of December–January and lowest in June–July [31]. This may not reflect the disease situation in the entire country. Therefore, it is difficult to draw any conclusions as the differences in sampling procedures may also affect conclusions [54]. Further studies may be necessary to better understand the seasonality of animal diseases in Bangladesh.

There is a routine anthrax, FMD, HS and PPR vaccination program for livestock in Bangladesh; however, the vaccination coverage was found to be very low (Table 1). The country has a capacity of annual production of only 3.4 million doses of anthrax vaccine, 0.4 million doses of bivalent (O, A) inactivated FMD vaccine, 0.8 million doses of formalin-killed HS vaccine and nearly 10 million doses of live attenuated PPR vaccine [55]. At present, Bangladesh is not producing any rabies vaccine. Tissue culture vaccine (TCV) is currently being imported from abroad mainly for humans [22]. For routine vaccination of livestock, DLS distributes locally produced animal vaccines (usually 100 ml/vial) at a subsidized rate of US$0.75 (50 taka). The abrupt decline in the numbers of anthrax cases in September–October in 2010 (Figure 2) could be due to production of immunity in many animals by massive vaccination by the government in particular areas (e.g., Sirajganj) of the country. Apparently, animals vaccinated against anthrax >6 months ago may not retain sufficient immunity resulting from the use of the attenuated Sterne strain vaccine in Bangladesh [56]. The efficacy of PPR vaccine is also a question due to its poor thermal stability and inadequate cold chain system in the country [57]. WHO recommends that 70% of a country’s dog population should be vaccinated to eliminate or prevent outbreaks of rabies [58]; however, rabies vaccinations in dogs or stray dog elimination programs are very limited especially in rural parts of Bangladesh [25]. Development of a protective level of immunity against any disease requires effective vaccines. As part of vaccination campaigns, DLS should assess vaccine efficacy, develop a strategy to improve coverage and conduct serosurveillance to determine coverage and duration of immunity. There is a need for more accurate means to evaluate the dog population and good rabies vaccination coverage. The current government program on modernization of livestock vaccine production is expected to dramatically increase the production of quality vaccines, and the vaccination program will be implemented in disease prevalent areas which will alter disease epidemiology.

Retrospective studies, based on data collected through passive surveillance, have contributed greatly in understanding animal disease epidemiology in Bhutan [28], India [48], [59] and Laos [60]. For a resource limited country like Bangladesh, passive surveillance can also play an important role in the overall surveillance system. However, the findings in this study need to be interpreted with caution because of the likely biases in the reporting system. First, although there has been no dramatic change in the surveillance for the diseases during the 2010 through 2012 study period, some minor differences in the reporting system may have occurred. Apparently, more monthly reports were missing (e.g., Rangamati and Feni districts) from 2010 than from 2011 or 2012. Some over-reporting of disease data (e.g., anthrax cases) may have occurred from some upazilas depending on the level of enthusiasm of the responsible livestock authority, which is not unexpected in a disease endemic country [61]. Second, some underreporting (e.g., anthrax deaths) may have occurred due to a lack of awareness of farmers. We assumed these possible variables remained relatively constant during the study period. Third, the number of laboratory confirmed cases was not available; this may not be critical since anthrax, FMD, HS, PPR and rabies cases are not difficult to diagnose in endemic countries based on clinical signs. However, some reporting biases may have been associated with cattle because buffaloes generally show no/mild signs of FMD and can serve as silent spreaders of the disease. The biases occurring from clinical diagnosis of the FMD in cattle are expected to be low because cattle are the predominant species in Bangladesh and clinical signs are more discernible in this species compared to other FMD-susceptible species [62]. Fourth, sometimes bite status may not be associated with rabies cases in livestock, as smallholding farmers who raise their livestock in public fields or rice fields may not know whether their cattle had been bitten by a rabid animal [63]. Owner’s interviews tend to be biased if the owners or their caretakers did not recognize abnormal changes in their animals. However, typical signs of rabies are very distinct and a misdiagnosis is unusual. The distinct late-stage clinical signs of rabies together with history of a dog bite mean that although misdiagnosis of other viral encephalitides remains a possibility, it is unlikely [64]. Fifth, the limited manpower restricts the ability of the veterinary authorities to respond to all reported cases. Introduction of web-based software, namely Livestock Disease Information System (LDIS), recently developed with the support from FAO ECTAD for reporting livestock diseases in Bangladesh, will enable tracking field cases of all diseases on a daily basis. Since use of the cell phone-Web based SMS-(Short Message Service) Gateway system has been successful with the surveillance of HPAI H5N1 in Bangladesh [65] and is popular nationally and internationally, this can also be of value for the surveillance of other important animal diseases.

In conclusion, this study provides information about the widespread distribution of anthrax, FMD, HS, PPR and dog bite/rabies in Bangladesh. The underlying factors could result from differences in the animal husbandry practices in different geographic locations, culture habits of animal uses, agro-climatic factors, lack of regulation of animal movements, lack of adequate vaccination and lack of knowledge about the epidemiology of the diseases. We recommend that the disease surveillance should be improved throughout the country, including a need for laboratory (serologic and molecular) confirmation of clinical cases. Probably, the most significant would be the identification and monitoring the circulation of the pathogens in the free-range and household animal populations. The findings from the present study constitute baseline data and provide the prompt for further investigation to better understand the epidemiology of important livestock diseases in Bangladesh.

## Supporting Information

Table S1 **District distribution of estimated number of diagnosed cases of anthrax, foot and mouth disease, haemorrhagic septicaemia, peste des petits ruminants and dog bite/rabies in livestock in Bangladesh, 2010–2012 (10 of the 64 districts with highest reported cases of each disease are highlighted as light grey for each year and as dark grey for three-year total).**
(DOCX)

Table S2 **Upazila distribution of estimated number of diagnosed cases of anthrax, foot and mouth disease, haemorrhagic septicaemia, peste des petits ruminants and dog bite/rabies in livestock in Bangladesh, 2010–2012 (6 of the 487 upazilas in the country with maximum reported cases of each disease are presented).**
(DOCX)

Table S3 **Geographic distribution (border** ***vs.*** **non-border districts) of estimated number of diagnosed cases and vaccination coverage of anthrax, foot and mouth disease, haemorrhagic septicaemia, peste des petits ruminants and dog bite/rabies in livestock in Bangladesh, 2010–2012.**
(DOCX)

Table S4 **Seasonal distribution of estimated number of diagnosed cases of anthrax, foot and mouth disease, haemorrhagic septicaemia, peste des petits ruminants and dog bite/rabies in livestock in Bangladesh, 2010–2012.**
(DOCX)
