# Supplementary material for: A Retrospective Study on the Epidemiology of Anthrax, Foot and Mouth Disease, Haemorrhagic Septicaemia, Peste des Petits Ruminants and Rabies in Bangladesh, 2010-2012
Source: PLoS One. 2014 Aug 7;9(8):e104435. doi: 10.1371/journal.pone.0104435 (PMC4125197; doi:10.1371/journal.pone.0104435)
Supplement: Table S1 — District distribution of estimated number of diagnosed cases of anthrax, foot and mouth disease, haemorrhagic septicaemia, peste des petits ruminants and dog bite/rabies in livestock in Bangladesh, 2010–2012 (10 of the 64 districts with highest reported cases of each disease are highlighted as light grey for each year and as dark grey for three-year total). (DOCX) [file pone.0104435.s001.docx]

Table S1

| **Sl.** |  |  |  | **Anthrax** |  |  |  | **FMD** |  |  |  | **HS** |  |  |  | **PPR** |  |  |  | **Rabies** |  |  |
| --- | --- | --- | --- | --- | --- | --- | --- | --- | --- | --- | --- | --- | --- | --- | --- | --- | --- | --- | --- | --- | --- | --- |
| **No.** | **Districts** | **Bordering** | **2010** | **2011** | **2012** | **Total** | **2010** | **2011** | **2012** | **Total** | **2010** | **2011** | **2012** | **Total** | **2010** | **2011** | **2012** | **Total** | **2010** | **2011** | **2012** | **Total** |
| 1 | Bagerhat | No | 153 | 87 | 29 | 269 | 246 | 795 | 1839 | 2880 | 12 | 28 | 20 | 60 | 2328 | 1782 | 1062 | 5172 | 71 | 44 | 131 | 246 |
| 2 | Bandarban | Yes | 64 | 0 | 0 | 64 | 309 | 200 | 0 | 509 | 64 | 169 | 0 | 233 | 160 | 143 | 0 | 303 | 4 | 1 | 0 | 5 |
| 3 | Barguna | No | 12 | 30 | 96 | 138 | 282 | 1094 | 2101 | 3477 | 0 | 450 | 427 | 877 | 419 | 850 | 2321 | 3590 | 16 | 12 | 0 | 28 |
| 4 | Barisal | No | 27 | 35 | 109 | 171 | 155 | 1880 | 5427 | 7462 | 0 | 183 | 25 | 208 | 97 | 778 | 2005 | 2880 | 12 | 62 | 64 | 138 |
| 5 | Bhola | No | 54 | 6 | 2 | 62 | 285 | 3025 | 3742 | 7052 | 53 | 349 | 90 | 492 | 88 | 715 | 1148 | 1951 | 41 | 29 | 90 | 160 |
| 6 | Bogra* | No | 74 | 4 | 8 | 86 | 1627 | 2386 | 3087 | 7100 | 59 | 90 | 76 | 225 | 4934 | 4346 | 4659 | 13939 | 124 | 202 | 223 | 549 |
| 7 | Brahmanbaria | Yes | 107 | 141 | 154 | 402 | 511 | 1941 | 4347 | 6799 | 0 | 0 | 69 | 69 | 297 | 1239 | 1655 | 3191 | 11 | 52 | 25 | 88 |
| 8 | Chandpur | No | 8 | 0 | 0 | 8 | 945 | 1871 | 3597 | 6413 | 3 | 38 | 30 | 71 | 127 | 405 | 642 | 1174 | 137 | 174 | 56 | 367 |
| 9 | Chittagong* | Yes | 48 | 85 | 115 | 248 | 3460 | 3939 | 7259 | 14658 | 127 | 150 | 225 | 502 | 1930 | 2488 | 3808 | 8226 | 59 | 76 | 169 | 304 |
| 10 | Chuadanga | Yes | 0 | 0 | 0 | 0 | 433 | 630 | 836 | 1899 | 4 | 20 | 7 | 31 | 959 | 1452 | 1531 | 3942 | 32 | 3 | 25 | 60 |
| 11 | Comilla* | Yes | 1 | 12 | 10 | 23 | 783 | 5347 | 12335 | 18465 | 2 | 116 | 69 | 187 | 517 | 1577 | 2752 | 4846 | 11 | 167 | 431 | 609 |
| 12 | Cox's Bazar | Yes | 8 | 5 | 106 | 119 | 1051 | 1952 | 3592 | 6595 | 64 | 87 | 108 | 259 | 400 | 857 | 1457 | 2714 | 31 | 152 | 160 | 343 |
| 13 | Dhaka | No | 33 | 0 | 0 | 33 | 844 | 2478 | 5497 | 8819 | 2 | 64 | 30 | 96 | 707 | 1683 | 1370 | 3760 | 19 | 25 | 62 | 106 |
| 14 | Dinajpur* | Yes | 16 | 0 | 0 | 16 | 753 | 1122 | 1508 | 3383 | 44 | 48 | 62 | 154 | 2725 | 2101 | 3662 | 8488 | 8 | 31 | 56 | 95 |
| 15 | Faridpur | No | 2 | 0 | 45 | 47 | 1459 | 1715 | 3420 | 6594 | 4 | 5 | 35 | 44 | 1065 | 1027 | 1091 | 3183 | 57 | 54 | 92 | 203 |
| 16 | Feni | Yes | 0 | 1 | 5 | 6 | 0 | 1533 | 375 | 1908 | 0 | 11 | 9 | 20 | 0 | 887 | 143 | 1030 | 0 | 115 | 89 | 204 |
| 17 | Gaibandha* | No | 36 | 6 | 20 | 62 | 199 | 600 | 336 | 1135 | 10 | 17 | 13 | 40 | 693 | 1090 | 1209 | 2992 | 99 | 120 | 320 | 539 |
| 18 | Gazipur | No | 11 | 0 | 0 | 11 | 342 | 1216 | 3915 | 5473 | 10 | 30 | 278 | 318 | 273 | 541 | 596 | 1410 | 11 | 46 | 559 | 616 |
| 19 | Gopalganj | No | 43 | 125 | 61 | 229 | 106 | 438 | 834 | 1378 | 6 | 112 | 0 | 118 | 275 | 504 | 45 | 824 | 6 | 12 | 6 | 24 |
| 20 | Habiganj | Yes | 0 | 48 | 103 | 151 | 8 | 984 | 4240 | 5232 | 0 | 20 | 23 | 43 | 333 | 324 | 1920 | 2577 | 0 | 25 | 89 | 114 |
| 21 | Jamalpur | Yes | 0 | 0 | 0 | 0 | 363 | 768 | 644 | 1775 | 9 | 105 | 11 | 125 | 425 | 755 | 812 | 1992 | 7 | 10 | 3 | 20 |
| 22 | Jessore* | Yes | 27 | 16 | 37 | 80 | 526 | 2527 | 4027 | 7080 | 69 | 55 | 62 | 186 | 3530 | 4434 | 7735 | 15699 | 77 | 124 | 272 | 473 |
| 23 | Jhalokathi | No | 0 | 14 | 119 | 133 | 94 | 39 | 4949 | 5082 | 0 | 138 | 71 | 209 | 179 | 122 | 441 | 742 | 0 | 46 | 24 | 70 |
| 24 | Jhenaidaha | Yes | 10 | 0 | 0 | 10 | 438 | 1056 | 2124 | 3618 | 1 | 342 | 12 | 355 | 873 | 1659 | 1527 | 4059 | 154 | 114 | 282 | 550 |
| 25 | Joypurhat | Yes | 4 | 0 | 0 | 4 | 323 | 627 | 1085 | 2035 | 3 | 74 | 0 | 77 | 1669 | 2249 | 3091 | 7009 | 9 | 93 | 194 | 296 |
| 26 | Khagrachhari | Yes | 8 | 10 | 0 | 18 | 126 | 76 | 352 | 554 | 27 | 0 | 0 | 27 | 82 | 5 | 89 | 176 | 10 | 3 | 8 | 21 |
| 27 | Khulna | No | 22 | 51 | 12 | 85 | 384 | 934 | 1023 | 2341 | 158 | 50 | 44 | 252 | 697 | 1756 | 631 | 3084 | 5 | 22 | 82 | 109 |
| 28 | Kishoreganj | No | 13 | 8 | 2 | 23 | 859 | 2513 | 5755 | 9127 | 24 | 21 | 47 | 92 | 897 | 1061 | 571 | 2529 | 66 | 79 | 109 | 254 |
| 29 | Kurigram* | Yes | 42 | 3 | 0 | 45 | 979 | 1209 | 686 | 2874 | 236 | 43 | 70 | 349 | 1073 | 864 | 1699 | 3636 | 119 | 105 | 80 | 304 |
| 30 | Kushtia | Yes | 28 | 2 | 0 | 30 | 506 | 858 | 987 | 2351 | 0 | 0 | 0 | 0 | 728 | 1374 | 681 | 2783 | 86 | 28 | 31 | 145 |
| 31 | Laksmipur | No | 1 | 0 | 0 | 1 | 423 | 453 | 801 | 1677 | 35 | 276 | 0 | 311 | 296 | 299 | 121 | 716 | 24 | 98 | 93 | 215 |
| 32 | Lalmonirhat | Yes | 11 | 0 | 0 | 11 | 183 | 1920 | 2250 | 4353 | 161 | 5 | 0 | 166 | 835 | 2184 | 5346 | 8365 | 13 | 62 | 135 | 210 |
| 33 | Madaripur | No | 6 | 2 | 1 | 9 | 606 | 767 | 959 | 2332 | 5 | 23 | 35 | 63 | 191 | 198 | 248 | 637 | 7 | 5 | 9 | 21 |
| 34 | Magura | No | 0 | 4 | 0 | 4 | 267 | 1292 | 1633 | 3192 | 51 | 19 | 36 | 106 | 960 | 1293 | 1476 | 3729 | 31 | 12 | 52 | 95 |
| 35 | Manikganj | No | 24 | 13 | 56 | 93 | 625 | 1486 | 2254 | 4365 | 28 | 129 | 132 | 289 | 1248 | 1314 | 1123 | 3685 | 118 | 54 | 20 | 192 |
| 36 | Meharpur | Yes | 1 | 0 | 0 | 1 | 412 | 376 | 1786 | 2574 | 0 | 6 | 56 | 62 | 753 | 1497 | 1737 | 3987 | 13 | 11 | 3 | 27 |
| 37 | Moulvibazar | Yes | 20 | 0 | 0 | 20 | 371 | 692 | 1688 | 2751 | 28 | 3 | 243 | 274 | 146 | 144 | 758 | 1048 | 7 | 14 | 83 | 104 |
| 38 | Munshiganj | No | 0 | 2 | 0 | 2 | 779 | 1586 | 4165 | 6530 | 37 | 19 | 7 | 63 | 282 | 641 | 1523 | 2446 | 273 | 80 | 330 | 683 |
| 39 | Mymensingh* | Yes | 41 | 3 | 5 | 49 | 1021 | 1946 | 2124 | 5091 | 49 | 8 | 26 | 83 | 2854 | 2483 | 3068 | 8405 | 85 | 79 | 337 | 501 |
| 40 | Naogaon* | Yes | 182 | 46 | 100 | 328 | 1263 | 1611 | 1933 | 4807 | 176 | 49 | 124 | 349 | 2240 | 2674 | 3975 | 8889 | 68 | 93 | 61 | 222 |
| 41 | Narail | No | 0 | 1 | 12 | 13 | 402 | 297 | 1249 | 1948 | 0 | 0 | 1 | 1 | 85 | 97 | 427 | 609 | 2 | 3 | 73 | 78 |
| 42 | Narayanganj | No | 41 | 205 | 0 | 246 | 1799 | 2764 | 2248 | 6811 | 17 | 272 | 40 | 329 | 1618 | 2053 | 386 | 4057 | 135 | 91 | 253 | 479 |
| 43 | Natore | No | 0 | 0 | 0 | 0 | 950 | 2113 | 2912 | 5975 | 21 | 5 | 66 | 92 | 2179 | 2468 | 1425 | 6072 | 16 | 121 | 53 | 190 |
| 44 | Nawabganj | Yes | 40 | 92 | 1 | 133 | 570 | 435 | 6232 | 7237 | 25 | 12 | 437 | 474 | 771 | 690 | 3432 | 4893 | 7 | 22 | 91 | 120 |
| 45 | Netrokona | Yes | 87 | 21 | 23 | 131 | 1045 | 2297 | 374 | 3716 | 108 | 154 | 42 | 304 | 1080 | 1175 | 2012 | 4267 | 98 | 134 | 16 | 248 |
| 46 | Nilphamari | Yes | 67 | 56 | 1 | 124 | 1118 | 2012 | 1606 | 4736 | 49 | 53 | 89 | 191 | 1331 | 1327 | 624 | 3282 | 27 | 0 | 184 | 211 |
| 47 | Nokhali | No | 43 | 0 | 10 | 53 | 641 | 901 | 765 | 2307 | 3 | 8 | 3 | 14 | 172 | 134 | 535 | 841 | 27 | 19 | 81 | 127 |
| 48 | Norsingdi | No | 78 | 1 | 0 | 79 | 926 | 1370 | 1355 | 3651 | 32 | 47 | 0 | 79 | 1126 | 1221 | 95 | 2442 | 18 | 27 | 54 | 99 |
| 49 | Pabna* | No | 11 | 0 | 0 | 11 | 959 | 1730 | 2246 | 4935 | 8 | 11 | 0 | 19 | 2297 | 1148 | 1397 | 4842 | 41 | 69 | 34 | 144 |
| 50 | Panchgarh | Yes | 27 | 10 | 10 | 47 | 226 | 579 | 223 | 1028 | 3 | 6 | 13 | 22 | 408 | 276 | 1800 | 2484 | 20 | 38 | 31 | 89 |
| 51 | Patuakhali | No | 32 | 40 | 24 | 96 | 587 | 2549 | 5337 | 8473 | 39 | 529 | 394 | 962 | 1042 | 923 | 581 | 2546 | 48 | 59 | 85 | 192 |
| 52 | Pirojpur | No | 13 | 44 | 0 | 57 | 201 | 171 | 788 | 1160 | 0 | 32 | 2 | 34 | 87 | 238 | 10 | 335 | 2 | 19 | 0 | 21 |
| 53 | Rajbari | No | 12 | 3 | 5 | 20 | 476 | 967 | 733 | 2176 | 3 | 3 | 14 | 20 | 1522 | 685 | 751 | 2958 | 46 | 21 | 44 | 111 |
| 54 | Rajshahi* | Yes | 33 | 0 | 0 | 33 | 3036 | 2342 | 1782 | 7160 | 75 | 141 | 69 | 285 | 6832 | 3645 | 3204 | 13681 | 67 | 40 | 94 | 201 |
| 55 | Rangmati | Yes | 0 | 13 | 202 | 215 | 0 | 105 | 833 | 938 | 0 | 12 | 104 | 116 | 0 | 59 | 659 | 718 | 0 | 26 | 70 | 96 |
| 56 | Rangpur* | No | 64 | 0 | 0 | 64 | 431 | 1253 | 500 | 2184 | 77 | 78 | 28 | 183 | 2875 | 1784 | 1983 | 6642 | 24 | 82 | 73 | 179 |
| 57 | Satkhira | Yes | 6 | 45 | 0 | 51 | 346 | 502 | 1059 | 1907 | 168 | 9 | 3 | 180 | 1404 | 1249 | 28 | 2681 | 61 | 21 | 1 | 83 |
| 58 | Shariatpur | No | 0 | 12 | 88 | 100 | 117 | 337 | 1687 | 2141 | 2 | 0 | 42 | 44 | 55 | 111 | 2163 | 2329 | 1 | 3 | 19 | 23 |
| 59 | Sherpur | Yes | 26 | 0 | 22 | 48 | 496 | 1186 | 1319 | 3001 | 78 | 0 | 1 | 79 | 369 | 689 | 849 | 1907 | 58 | 46 | 103 | 207 |
| 60 | Sirajganj* | No | 111 | 0 | 0 | 111 | 2033 | 2925 | 1761 | 6719 | 111 | 191 | 129 | 431 | 1874 | 2120 | 580 | 4574 | 161 | 301 | 454 | 916 |
| 61 | Sunamganj | Yes | 59 | 85 | 180 | 324 | 462 | 1433 | 7761 | 9656 | 18 | 131 | 263 | 412 | 229 | 403 | 2421 | 3053 | 33 | 46 | 255 | 334 |
| 62 | Sylhet | Yes | 58 | 13 | 177 | 248 | 1436 | 2162 | 5580 | 9178 | 69 | 158 | 141 | 368 | 936 | 1048 | 1359 | 3343 | 1 | 48 | 96 | 145 |
| 63 | Tangail* | No | 11 | 0 | 0 | 11 | 1304 | 2961 | 2884 | 7149 | 29 | 39 | 8 | 76 | 1209 | 1232 | 1068 | 3509 | 82 | 79 | 99 | 260 |
| 64 | Thakurgaon* | Yes | 218 | 268 | 145 | 631 | 407 | 2695 | 1305 | 4407 | 286 | 642 | 308 | 1236 | 1898 | 1871 | 2141 | 5910 | 34 | 55 | 133 | 222 |
|  |  | **Total=** | **2174** | **1668** | **2095** | **5937** | **44314** | **93968** | **162051** | **300333** | **2782** | **5885** | **4769** | **13436** | **69684** | **78441** | **99658** | **247783** | **2930** | **3904** | **7251** | **14085** |

*District with proportionately higher livestock (cattle, goats, sheep and buffaloes) population
