# Supplementary material for: A Retrospective Study on the Epidemiology of Anthrax, Foot and Mouth Disease, Haemorrhagic Septicaemia, Peste des Petits Ruminants and Rabies in Bangladesh, 2010-2012
Source: PLoS One. 2014 Aug 7;9(8):e104435. doi: 10.1371/journal.pone.0104435 (PMC4125197; doi:10.1371/journal.pone.0104435)
Supplement: Table S2 — Upazila distribution of estimated number of diagnosed cases of anthrax, foot and mouth disease, haemorrhagic septicaemia, peste des petits ruminants and dog bite/rabies in livestock in Bangladesh, 2010–2012 (6 of the 487 upazilas in the country with maximum reported cases of each disease are presented). (DOCX) [file pone.0104435.s002.docx]

Table S2

|  | **2010** |  |  | **2011** |  |  | **2012** |  |
| --- | --- | --- | --- | --- | --- | --- | --- | --- |
| **Disease** | **Sub-district (District)** | **Diagnosed cases (%)** |  | **Sub-district (District)** | **Diagnosed cases (%)** |  | **Sub-district (District)** | **Diagnosed cases (%)** |
| Anthrax | Ranishankail (Thakurgaon) | 105 (4.83) |  | Sonargaon (Narayanganj) | 183 (10.97) |  | Belaichhari (Rangamati) | 165 (7.88) |
|  | Rampal (Bagerhat) | 104 (4.78) |  | Haripur (Thakurgaon) | 142 (8.51) |  | Sarail (Brahmanbaria) | 154 (7.35) |
|  | Sarail (Brahmanbaria) | 100 (4.60) |  | Sarail (Brahmanbaria) | 130 (7.79) |  | Haripur (Thakurgaon) | 133 (6.35) |
|  | Patnitala (Naogaon) | 95 (4.37) |  | Kotalipara (Gopalganj) | 125 (7.49) |  | Jhalokati Sadar (Jhalokati) | 119 (5.68) |
|  | Jaldhaka (Nilphamari) | 67 (3.08) |  | Nachole (Nawabganj) | 86 (5.16) |  | Doarabazar (Sunamganj) | 119 (5.68) |
|  | Belkuchi (Sirajganj) | 56 (2.58) |  | Dowarabazar (Sunamganj) | 82 (4.92) |  | Fenchuganj (Sylhet) | 115 (5.49) |
|  | **Total=** | **527 (24.24)** |  | **Total=** | **748 (44.84)** |  | **Total=** | **805 (38.43)** |
| FMD | Charghat (Rajshahi) | 1664 (3.76) |  | Kaliganj (Lalmonirhat) | 1493 (1.59) |  | Nalchhiti (Jhalokati) | 4830 (2.98) |
|  | Boalkhali (Chittagong) | 832 (1.88) |  | Muradnagar (Comilla) | 1471 (1.57) |  | Doarabazar (Sylhet) | 4388 (2.71) |
|  | Jaldhaka (Nilphamari) | 758 (1.71) |  | Ullapara (Sirajganj) | 1429 (1.52) |  | Nikli (Kishoreganj) | 3496 (2.16) |
|  | Muradnagar (Comilla) | 650 (1.47) |  | Nangalkot (Comilla) | 1092 (1.16) |  | Gournadi (Barisal) | 2805 (1.73) |
|  | Ullapara (Sirajganj) | 631 (1.42) |  | Pirganj (Thakurgaon) | 1078 (1.15) |  | Baraigram (Natore) | 2479 (1.53) |
|  | Panchalaish (Chittagong) | 602 (1.36) |  | Gaurnadi (Barisal) | 950 (1.01) |  | Muradnagar (Comilla) | 2245 (1.39) |
|  | **Total=** | **5137 (11.59)** |  | **Total=** | **7513 (8.00)** |  | **Total=** | **20243 (12.49)** |
| HS | Ranishankail (Thakurgaon) | 232 (8.34) |  | Patuakhali Sadar (Patuakhali) | 373 (6.34) |  | Baraigram (Natore) | 434 (9.10) |
|  | Patgram (Lalmonirhat) | 150 (5.39) |  | Barguna Sadar (Barguna) | 372 (6.32) |  | Barguna Sadar (Barguna) | 427 (8.95) |
|  | Rajibpur (Kurigram) | 117 (4.21) |  | Haripur (Thakurgaon) | 371 (6.30) |  | Patuakhali Sadar (Patuakhali) | 374 (7.84) |
|  | Porsha (Naogaon) | 100 (3.60) |  | Shailkupa (Jhenaidah) | 338 (5.74) |  | Kulaura (Moulavibazar) | 238 (4.99) |
|  | Satkhira Sadar (Satkhira) | 95 (3.42) |  | Ranisankail (Thakurgaon) | 271 (4.61) |  | Kapasia (Gazipur) | 261 (5.47) |
|  | Shahajadpur (Sirajganj) | 75 (2.70) |  | Sonargaon (Narayanganj) | 270 (4.59) |  | Ranishankail (Thakurgaon) | 192 (4.03) |
|  | **Total=** | **769 (27.64)** |  | **Total=** | **1995 (33.90)** |  | **Total=** | **1926 (40.39)** |
| PPR | Badarganj (Rangpur) | 1391 (2.00) |  | Sonargaon (Narayanganj) | 1459 (1.86) |  | Lalmonirhat Sadar (Lalmonirhat) | 2790 (2.80) |
|  | Charghat (Rajshahi) | 1348 (1.93) |  | Gangni (Meherpur) | 1304 (1.66) |  | Abhaynagar (Jessore) | 2542 (2.55) |
|  | Boalia (Rajshahi) | 1195 (1.72) |  | Ullapara (Sirajganj) | 1294 (1.65) |  | Sarsha (Jessore) | 2523 (2.53) |
|  | Jessore Sadar ( Jessore) | 1055 (1.51) |  | Sharsha (Jessore) | 1051 (1.34) |  | Lalpur (Natore) | 1599 (1.60) |
|  | Paba (Rajshahi) | 999 (1.43) |  | Dighalia (Khulna) | 1042 (1.33) |  | Joypurhat Sadar (Joypurhat) | 1445 (1.45) |
|  | Dimla (Nilphamari) | 943 (1.35) |  | Mymensingh Sadar (Mymensingh) | 1025 (1.31) |  | Paba (Rajshahi) | 1434 (1.44) |
|  | **Total=** | **6931 (9.95)** |  | **Total=** | **7175 (9.15)** |  | **Total=** | **12333 (12.38)** |
| Dog bite | Lahajang (Munshiganj) | 240 (8.19) |  | Shahjadpur (Sirajganj) | 213 (5.46) |  | Shahjadpur (Sirajganj) | 329 (4.54) |
| /rabies | Shailkupa (Jhenaidah) | 125 (4.27) |  | Muradnagar (Comilla) | 106 (2.72) |  | Lohajang (Munshiganj) | 325 (4.48) |
|  | Shahjadpur (Sirajganj) | 99 (3.38) |  | Ramganj (Lakshmipur) | 96 (2.46) |  | Gazipur Sadar (Gazipur) | 292 (4.03) |
|  | Ghior (Manikganj) | 87 (2.97) |  | Dhupchanchia (Bogra) | 95 (2.43) |  | Gaibandha Sadar (Gaibandha) | 196 (2.70) |
|  | Palasbari (Gaibandha) | 73 (2.49) |  | Chandpur Sadar (Chandpur) | 86 (2.20) |  | Jheneidah Sadar (Jheneidah) | 185 (2.55) |
|  | Chandpur Sadar (Chandpur) | 69 (2.36) |  | Lahajang (Munshiganj) | 78 (2.00) |  | Kapasia (Gazipur) | 183 (2.52) |
|  | **Total=** | **693 (23.65)** |  | **Total=** | **674 (17.26)** |  | **Total=** | **1510 (20.83)** |
