# Supplementary material for: A Retrospective Study on the Epidemiology of Anthrax, Foot and Mouth Disease, Haemorrhagic Septicaemia, Peste des Petits Ruminants and Rabies in Bangladesh, 2010-2012
Source: PLoS One. 2014 Aug 7;9(8):e104435. doi: 10.1371/journal.pone.0104435 (PMC4125197; doi:10.1371/journal.pone.0104435)
Supplement: Table S3 — Geographic distribution (border vs. non-border districts) of estimated number of diagnosed cases and vaccination coverage of anthrax, foot and mouth disease, haemorrhagic septicaemia, peste des petits ruminants and dog bite/rabies in livestock in Bangladesh, 2010–2012. (DOCX) [file pone.0104435.s003.docx]

Table S3

|  |  | **2010** |  |  | **2011** |  |  | **2012** |  |  | **Total** |  |  |
| --- | --- | --- | --- | --- | --- | --- | --- | --- | --- | --- | --- | --- | --- |
| **Disease**ᵟ | **District**  **group**ᶿ | **Diagnosed cases (Prevalence, %)** | **Vaccination (Vac. rate**‡**, %)** |  | **Diagnosed cases (Prevalence, %)** | **Vaccination (Vac. rate, %)** |  | **Diagnosed cases (Prevalence, %)** | **Vaccination (Vac. rate, %)** |  | **Diagnosed cases (Prevalence, %)** | **Vaccination (Vac. rate, %)** |  |
| Anthrax | Border | 1239 (0.16)**ǂ** | 1139128 (4.64)* |  | 975 (0.10)ᶿ | 1607486 (6.55)* |  | 1396 (0.22) | 1776563 (7.23) |  | 3610 (0.15) | 4523177 (6.14)* |  |
|  | Non-border | 935 (0.12) | 1463839 (8.12) |  | 693 (0.07) | 1809650 (10.03) |  | 699 (0.11) | 1548962 (8.59) |  | 2327 (0.10) | 4822451 (8.91) |  |
| FMD | Border | 22961 (2.91) | 113225 (0.46)* |  | 22961 (5.00) | 89982 (0.37)* |  | 82252 (13.04) | 134779 (0.55)* |  | 152275 (6.45) | 337986 (0.46)* |  |
|  | Non-border | 21353 (2.72) | 139859 (0.78) |  | 21353 (4.91) | 123672 (0.69) |  | 79799 (12.78) | 176939 (0.98) |  | 148058 (6.26) | 440470 (0.81) |  |
| HS | Border | 1943 (0.25) | 145846 (0.59) |  | 2629 (0.28) | 152036 (0.62)* |  | 2646 (0.42) | 212256 (0.86)* |  | 7218 (0.31) | 510138 (0.69)* |  |
|  | Non-border | 839 (0.11) | 126549 (0.70) |  | 3256 (0.34) | 215836 (1.20) |  | 2123 (0.34) | 220845 (1.22) |  | 6218 (0.26) | 563230 (1.04) |  |
| PPR | Border | 37787 (12.08)† | 938708 (8.94) |  | 43822 (12.15)ᵟ | 1388587 (13.23) |  | 65975 (28.79) | 1527788 (14.56) |  | 147584 (16.35) | 3855083 (12.24) |  |
|  | Non-border | 31897 (12.07) | 575768 (8.27) |  | 34619 (10.33) | 739112 (10.62) |  | 33683 (18.13) | 899860 (12.93) |  | 100199 (12.76) | 2214740 (10.60) |  |
| Dog bite | Border | 1210 (0.15) | 272 (22.48) |  | 1834 (0.20) | 300 (16.36) |  | 3607 (0.57) | 700 (19.41) |  | 6651 (0.28) | 1316 (19.68) |  |
| /rabies | Non-border | 1720 (0.22) | 307 (17.85) |  | 2070 (0.22) | 467 (22.56) |  | 3644 (0.58) | 934 (25.63) |  | 7434 (0.31) | 1758 (23.51) |  |

ᵟAbbreviated: FMD = Foot and mouth disease, HS = Haemorrhagic septicaemia, PPR = Peste des petits ruminants

ᶿBorder vs. non-border: 50% of districts (32/64) of the country bordering India (31) and Myanmar (1)

**ǂ**Based on number of livestock (buffaloes, cattle, goats and sheep) examined: border (n=790,126) *vs.* non-border (n=786,319) districts in 2010; border (n=941,384) *vs.* non-border (n= 954,922) districts in 2011; and border (n=630,815) *vs.* non-border (n= 624,330) districts in 2012

†Based on number of goats and sheep examined: border (n=312,703) *vs.* non-border (n=264,273) districts in 2010; border (n=360,555) *vs.* non-border (n=335,040) districts in 2011; and border (n=229,202) *vs.* non-border (n=185,805) districts in 2012

‡Based on susceptible livestock (buffaloes, cattle, goats and sheep) population in Bangladesh [5]: border (n=24,558,339) *vs.* non-border (n=18,036,060) districts; for PPR, goats and sheep population only: border (n=10,496,964) *vs.* non-border (n=6,962,097) districts; for rabies, the post-exposure vaccination was calculated based on the reported dog bite cases

*Statistically significant at the p<0.01 level
