# Supplementary material for: A Retrospective Study on the Epidemiology of Anthrax, Foot and Mouth Disease, Haemorrhagic Septicaemia, Peste des Petits Ruminants and Rabies in Bangladesh, 2010-2012
Source: PLoS One. 2014 Aug 7;9(8):e104435. doi: 10.1371/journal.pone.0104435 (PMC4125197; doi:10.1371/journal.pone.0104435)
Supplement: Table S4 — Seasonal distribution of estimated number of diagnosed cases of anthrax, foot and mouth disease, haemorrhagic septicaemia, peste des petits ruminants and dog bite/rabies in livestock in Bangladesh, 2010–2012. (DOCX) [file pone.0104435.s004.docx]

Table S4

|  |  | **2010** |  |  | **2011** |  |  | **2012** |  |  | **Total** |  |
| --- | --- | --- | --- | --- | --- | --- | --- | --- | --- | --- | --- | --- |
| **Disease**ᵟ | **Seasonǂ** | **Diagnosed**  **cases (%)** | **Death cases (CFR, %)** |  | **Diagnosed**  **cases (%)** | **Death cases (CFR, %)** |  | **Diagnosed**  **cases (%)** | **Death cases (CFR, %)** |  | **Diagnosed**  **cases (%)** | **Death cases (CFR, %)** |
| Anthrax | Pre-monsoon | 507 (23.3) | 115 (22.7) |  | 354 (21.2) | 40 (11.3) |  | 598 (28.5)* | 56 (9.4) |  | 1459 (24.6) | 211 (14.5) |
|  | Monsoon | 926 (42.6)* | 178(19.2) |  | 529 (31.7)* | 54 (10.2) |  | 562 (26.8)* | 39 (6.9) |  | 2017 (34.0)* | 271 (13.4) |
|  | Post-monsoon | 271 (12.5) | 66 (24.4) |  | 458 (27.5) | 44 (9.6) |  | 487 (23.3) | 50 (10.3) |  | 1216 (20.5) | 160 (13.2) |
|  | Winter | 470 (21.6) | 74 (15.8) |  | 327 (19.6) | 35(10.7) |  | 448 (21.4) | 50 (11.2) |  | 1245 (21.0) | 159 (12.8) |
| FMD | Pre-monsoon | 9148 (20.6) | 167 (1.8) |  | 28942 (30.8)* | 539 (1.9) |  | 42150 (26.0) | 621 (1.5) |  | 80240 (26.7) | 1327 (1.7) |
|  | Monsoon | 10303 (23.3) | 161 (1.6) |  | 26498 (28.2)* | 438 (1.7) |  | 42304 (26.1) | 375 (0.9) |  | 79105 (26.3) | 974 (1.2) |
|  | Post-monsoon | 13105 (29.6)* | 156 (1.5) |  | 19927 (21.2) | 293 (1.7) |  | 45101 (27.8) | 408 (0.9) |  | 78133 (26.0) | 857 (1.1) |
|  | Winter | 11758 (26.5)* | 167 (1.4) |  | 18601 (19.8) | 296 (1.6) |  | 32496 (20.1) | 317 (1.0) |  | 62855 (20.9) | 780 (1.2) |
| HS | Pre-monsoon | 703 (25.3) | 22 (3.1) |  | 1083 (18.4) | 20 (1.9) |  | 1086 (22.8) | 50 (4.6) |  | 2872 (21.4) | 92 (3.2 ) |
|  | Monsoon | 704 (25.3) | 11 (1.6) |  | 1112 (18.9) | 20 (1.8) |  | 1132 (23.7) | 21 (1.9) |  | 2948 (21.9) | 52 (1.8) |
|  | Post-monsoon | 807 (29.0)* | 27 (3.4) |  | 2391 (40.6)* | 162 (6.8)* |  | 1365 (28.6)* | 17 (1.3) |  | 4563 (34.0)* | 206 (4.5)* |
|  | Winter | 568 (20.4) | 4 (0.7) |  | 1299 (22.1) | 19 (1.5) |  | 1186 (24.9) | 13 (1.1) |  | 3053 (22.7) | 36 (1.2) |
| PPR | Pre-monsoon | 15605 (22.4) | 1161 (7.4) |  | 20967 (26.7)* | 1064 (5.1) |  | 23598 (23.7) | 937 (4.0) |  | 60170 (24.3) | 3162 (5.3) |
|  | Monsoon | 20557 (29.5)* | 1272 (6.2) |  | 21368 (27.2)* | 1171 (5.5) |  | 26944 (27.0)* | 844 (3.1) |  | 68869 (27.8)* | 3287 (4.8) |
|  | Post-monsoon | 19302 (27.7) | 1054 (5.5) |  | 17862 (22.8) | 846 (4.7) |  | 27691 (27.8)* | 952 (3.4) |  | 64855 (26.2) | 2852 (4.4) |
|  | Winter | 14220 (20.4) | 930 (6.5) |  | 18244 (23.3) | 1013 (5.6) |  | 21425 (21.5) | 790 (3.7) |  | 53889 (21.8) | 2733 (5.1) |
| Dog bite | Pre-monsoon | 615 (21.0) | 161 (26.2) |  | 611 (15.7) | 201(32.9) |  | 1545 (21.1) | 390 (25.2) |  | 2771 (19.6) | 752 (27.1) |
| /rabies | Monsoon | 480 (16.4) | 143 (29.8) |  | 1090 (27.9) | 323 (29.6) |  | 2205 (30.1)* | 373 (16.9) |  | 3775 (26.7) | 839 (22.2) |
|  | Post-monsoon | 835 (28.5)* | 187 (22.4) |  | 1185 (30.4)* | 343 (29.0) |  | 2000 (27.3)* | 431 (21.6) |  | 4020 (28.4)* | 961 (23.9) |
|  | Winter | 1000 (34.1)* | 250 (25.0) |  | 1018 (26.1)* | 291 (28.6) |  | 1501 (20.5) | 291 (19.4) |  | 3519 (24.8) | 832 (23.6) |

ᵟAbbreviated: FMD = Foot and mouth disease, HS = Haemorrhagic septicaemia, PPR = Peste des petits ruminants

ǂEach year was divided into the four main weather seasons of Bangladesh [36]: pre-monsoon (March to May), monsoon (June to August), post-monsoon (September to November) and winter (December to February)
